# Effect of electroconvulsive therapy on brain‐derived neurotrophic factor levels in patients with major depressive disorder

**DOI:** 10.1002/brb3.1101

**Published:** 2018-10-01

**Authors:** Annamari Sorri, Kaija Järventausta, Olli Kampman, Kai Lehtimäki, Minna Björkqvist, Kati Tuohimaa, Mari Hämäläinen, Eeva Moilanen, Esa Leinonen

**Affiliations:** ^1^ Department of Psychiatry Tampere University Hospital Tampere Finland; ^2^ Department of Psychiatry, School of Medicine University of Tampere Tampere Finland; ^3^ Department of Psychiatry Seinäjoki Hospital District Seinäjoki Finland; ^4^ Department of Neurosurgery, Neurology and Rehabilitation Tampere University Hospital Tampere Finland; ^5^ The Immunopharmacology Research Group, Faculty of Medicine and Life Sciences University of Tampere and Tampere University Hospital Tampere Finland

**Keywords:** brain‐derived neurotrophic factor, electroconvulsive therapy, major depressive disorder, neurotrophin

## Abstract

**Objectives:**

Brain‐derived neurotrophic factor (BDNF) has been associated with depression and its treatment response. The aim of the present study was to explore the effect of electroconvulsive therapy (ECT) on serum and plasma BDNF levels and change of Montgomery–Asberg Depression Rating Scale (MADRS) and their associations in patients with major depressive disorder (MDD).

**Methods:**

The study included thirty patients suffering from MDD. Their serum and plasma BDNF levels were examined before ECT (baseline) and after the first, fifth, and last ECT session. The severity of the depression and the response to ECT were measured with MADRS.

**Results:**

Electroconvulsive therapy caused no significant changes in serum BDNF levels. Plasma BDNF levels decreased during the fifth ECT session between the baseline and the 2‐hr samples (*p* = 0.019). No associations were found between serum or plasma BDNF levels and remission. The correlations between plasma and serum BDNF levels in each measurement varied between 0.187 and 0.636.

**Conclusions:**

Neither serum nor plasma BDNF levels were systematically associated with the clinical remission. However, the plasma BDNF levels somewhat varied during the ECT series. Therefore, the predictive value of BDNF for effects of ECT appears to be at least modest.

## 1 INTRODUCTION

Electroconvulsive therapy (ECT) is considered to be the most effective therapy for severe major depressive disorder (MDD) with reported effectiveness rates between 80% and 90% (UK ECT Review Group, 2003). Indication of ECT as the first‐line treatment is a need for rapid, definitive response because of the severity of the psychotic or suicidal symptoms as well as a favorable prior response to ECT. Treatment resistance is a secondary indication for ECT (American Psychiatric Association, 2001). ECT is well tolerated; for example, it does not cause long‐term cognitive impairment (Haghighi et al., 2016; Haghighi, Bajoghli, et al., 2013).

Molecules in several brain areas are affected by ECT including neurotransmitters, neuropeptides, and neurotrophic factors (Wahlund & von Rosen, 2003). It has been demonstrated that electroconvulsive stimulation (ECS), an animal model of ECT, induced neurogenesis in rat hippocampus suggesting that the effect of ECT may be related to the stimulation of cellular and synaptic plasticity in the hippocampal formation (Madsen et al., 2000).

Brain‐derived neurotrophic factor (BDNF) is a member of the neurotrophin family of trophic factors and is abundantly expressed in the central nervous system, especially in the hippocampus and cerebral cortex (Altar, 1999; Leibrock et al., 1989; Lewin & Barde, 1996). BDNF is involved in stimulating the development and differentiation of new neurons, neuron survival, and promoting long‐term potentiation (Noble, Billington, Kotz, & Wang, 2011). Molendijk et al. (2014) concluded in a meta‐analysis that serum BDNF levels were reduced in patients suffering from MDD and that the BDNF levels were elevated following a course of antidepressant drug treatment (Molendijk et al., 2014).

Increased BDNF expression has been reported to mediate the antidepressant effects of a range of antidepressants as well as ECS in some animal studies (Castrén & Rantamäki, 2010). Levels of BDNF have been reported to rise in various parts of rodent brain after ECS (Angelucci, Aloe, Jimenez‐Vasquez, & Mathe, 2002), (Altar, Whitehead, Chen, Wörtwein, & Madsen, 2003). However, there is also an opposite finding observing no change in BDNF concentration in rodent brain areas after ECS (Angelucci, Aloe, Jimenez‐Vasquez, & Mathe, 2003). Secretion of the precursor of BDNF, proBDNF, was recently found to be increased in rat hippocampus after a single administration of ECS. After repeated ECS, accumulation of proBDNF resulted in an increase in BDNF (Segawa, Morinobu, Matsumoto, Fuchikami, & Yamawaki, 2013). However, the importance of the rodent forced swim stressor test (FST) used as a depression model in studies concerning the pathogenesis and treatment of mood disorders has been questioned. In a recent review article, the conclusion was that the rodent's behavioral response to FST reflects stress coping and adaptation mechanisms rather than depression (De Kloet & Molendijk, 2016).

In human studies, the findings on the changes in BDNF and the response to ECT are controversial (Pinna et al., 2016). Some studies have suggested that ECT increases serum or plasma BDNF levels at different time points after ECT (Bilgen et al., 2014; Bocchio‐Chiavetto et al., 2006; Bumb et al., 2014; Haghighi, Salehi, et al., 2013; Hu et al., 2010; Marano et al., 2007; Okamoto et al., 2008; Piccinni et al., 2009; Salehi et al., 2016). However, there are several studies which have reported no influence of ECT on serum or plasma BDNF levels during or after ECT series (Fernandes et al., 2009; Gedge et al., 2012; Grønli, Stensland, Wynn, & Olstad, 2009; Kleimann et al., 2014; Lin et al., 2013; Rapinesi et al., 2015). These studies examined either serum or plasma levels of BDNF mainly before and after ECT series. It is not yet understood whether these findings reflect the processes in central nervous system during ECT.

The aim of the present study was to examine the predictive effect of serum and plasma BDNF levels on remission in patients with MDD at three stages during the ECT series. The three stages were designed to examine the immediate impact of ECT on serum and plasma levels of BDNF at specific time points during ECT series. Also, the aim was to find out whether the findings between serum and plasma BDNF levels were consistent with each other.

## 2 PATIENTS AND METHODS

The study group consisted of 36 eligible patients (17 females and 19 male) consecutively admitted for ECT to the Department of Psychiatry, Tampere University Hospital. Six patients withdrew from the study, so that the final study population consisted of 30 patients (12 females and 18 male). The patients were recruited between November 2011 and May 2013. On admission, the structured clinical interview for DSM‐IV disorders interview (SCID) (First, Spitzer, Gibbon, & Williams, 1996) was conducted with each patient to confirm the diagnosis. All patients fulfilled the DSM‐IV diagnostic criteria for major depressive disorder (MDD) (American Psychiatric Association, 1994), and 14 of them had psychotic symptoms. Nine patients had suffered their first episode of MDD, and 21 patients were suffering from recurrent MDD.

Patients with progressive organic brain disorder, other major psychiatric disorder than MDD, inflammatory or autoimmune disease, epilepsy, and alcohol or other substance abuse were excluded from the study. Patients who had received ECT within 3 months prior to entry into the study were also excluded.

The mean age of the patients was 57.1 years (*SD* 17.7, range 25–85 years). The patients were given constant psychotropic drug treatment throughout the entire ECT period (Table 1). Twenty‐eight patients in the study were taking a combination of at least two psychotropic medications, and only two were on monotherapy. Benzodiazepines were discontinued 10 hr before ECT to avoid effects on seizure threshold.

**Table 1:** Psychotropic medications of patients with MDD during the ECT series

	All patients (*n* = 30)	Female patients (*n* = 12)	Male patients (*n* = 18)
Age, mean, ±*SD* range	57.1, ±17.7 25–85	71.1 ± 12.2 45–85	45.2 ± 17.0 25–79
Total number of ECTs, mean, ±*SD* range	10.4, ±3.6 5–17	10.8 ± 4.3 5–17	8.2 ± 3.6 5–13
Psychotic symptoms	14	7	7
First episode of MDD	9	4	5
Recurrent MDD	21	13	8
Antidepressants			
SSRI	9	3	6
SNRI	12	5	7
Mirtazapine	12	6	6
Bupropion	4	1	3
Antipsychotics			
Second‐generation antipsychotics	28	11	17
Conventional neurolepts	1	0	1
Anxiolytics			
Benzodiazepines	21	8	13
Pregabalin	2	2	0
Buspirone	1	0	1

Note

SNRI: serotonin and norepinephrine reuptake inhibitor; SSRI: Selective serotonin reuptake inhibitor.

The severity of the depression was quantified by the Montgomery–Asberg Depression Rating Scale (MADRS) (Montgomery & Åsberg, 1979) before the first and after the fifth and last ECT.

Before the first ECT treatment, a medical history, a physical examination with routine blood examinations, and end electrocardiogram (ECG) were requested. ECT was administered three times a week with a brief pulse constant current device MECTA SPECTRUM 5000Q (MECTA Corp., Lake Oswego, OR, USA). Seizure threshold was titrated at treatment 1, and subsequent treatments were administered at 1.5 times the seizure threshold for bilateral (BL) ECT. Seizures over 25 s in duration in EEG were deemed adequate.

Anesthesia was induced with methohexital and muscle relaxation with succinylcholine. The initial dose was 1 mg/kg of methohexital and 0.5 mg/kg of succinylcholine. The patients were ventilated with 100% oxygen until resumption of spontaneous respiration. Physiological monitoring during the treatment included pulse oximetry, blood pressure, ECG, one‐channel electroencephalogram (EEG), and electromyography (EMG). All the patients were treated with standard bilateral (bifrontotemporal) ECT. The number of treatments ranged between 5 and 17, 10.4 ± 3.6 (mean, ±*SD*). ECT treatment was discontinued on the basis of clinical judgment if the patient was either in remission (MADRS ≤ 10) or no further improvement was recorded during last two ECT sessions. In five patients, the fifth ECT was the last (not included in last ECT session data).

Blood was drawn before ECT (baseline) and 2 hr and 4 hr after ECT at the first, fifth, and last session, and EDTA plasma/serum (P/S) was separated and stored at −80 C until analyzed. The concentration of BDNF in plasma/serum samples was determined using enzyme‐linked immunosorbent assay (DuoSet ELISA; R&D Systems Europe, Ltd, Abingdon, UK).

All patients gave written informed consent. This study design was reviewed and approved by the Tampere University Hospital Ethics Committee.

### 2.1 Statistical methods

Differences in serum BDNF or plasma BDNF levels between different measurements (baseline; after 2 hr and after 4 hr at the first, fifth, and last ECT treatment sessions; and between baseline levels of first, fifth, and last treatment sessions) were calculated with repeated samples ANOVA. Pearson correlations were used for comparisons between simultaneous S‐BDNF and P‐BDNF levels in each measurement. Due to nonnormal distributions, logarithmic transformations were used for P‐BDNF measurements in all analyses. The level of statistical level was set at *p* < 0.05. All significant differences are also reported as effect sizes (Cohen's *d*). Power analysis showed that, with the current sample, a difference of 1.89 ng/ml in serum BDNF levels between paired samples was detectable with a power of 0.8. Calculations were performed with R (version 3.2.0), SPSS for Windows (version 22.0; IBM Inc.), and PS Power and Sample size calculator software (Dupont & Plummer, 1990).

## 3 RESULTS

At baseline, the mean MADRS score was 31.6 ± 7.2 (mean ± *SD*), and after ECT series, the mean score was 11.3 ± 7.5. At the end of the study, 20 of 30 patients were in remission (MADRS ≤ 10).

**Figure 1:**
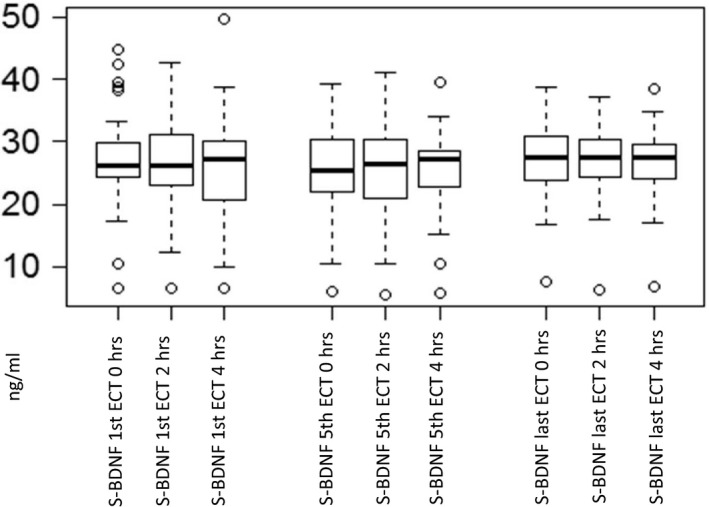
Serum BDNF levels in the first, fifth, and last ECT sessions

Electroconvulsive therapy caused no significant changes in serum BDNF levels (mean levels 25.1–27.3 ng/ml) between and during the first, fifth, and last ECT sessions (Figure 1). There was no correlation between baseline (before the first ECT) serum BDNF and severity of depression measured by MADRS. Baseline serum BDNF did not predict the later remission rate after the treatment period.

Plasma BDNF concentrations of the present patients were around one‐fourth compared to serum levels (variation 6.1–11.4 ng/ml). More variation in plasma BDNF was seen than in serum levels. The correlations between plasma and serum BDNF levels in each measurement varied between 0.187 and 0.636 (Pearson correlation coefficient).

Plasma BDNF levels fell during the fifth ECT session between baseline and the 2‐hr samples (*p* = 0.038, *d* = 0.45). No significant changes were found in plasma BDNF levels during the first ECT session, but there was a decreasing trend between baseline and 2‐hr plasma BDNF levels during the last ECT session (*p* = 0.079, *d* = 0.42). No associations were found between any plasma BDNF levels and their changes and remission.

## 4 DISCUSSION

In the present study, serum BDNF levels were not influenced by ECT. In plasma, the BDNF levels decreased during the fifth ECT session between the baseline and the 2‐hr samples (*p* = 0.019). Serum and plasma BDNF levels appeared to be inconsistent with each other, and the correlations between plasma and serum BDNF levels in each measurement varied between 0.187 and 0.636. The variations in plasma BDNF levels were large.

The primary aim of this study was to examine the influence of ECT on serum and plasma BDNF levels during ECT series and whether the levels or change there would act as a predictive factor for remission. The study was targeted to examine these processes during the single ECT session and throughout the ECT series. To accomplish these aims, the measurement points were defined before ECT (baseline) and 2 and 4 hr after ECT at the first, fifth, and last session. Since the methodology of the previous studies concerning the effects of ECT on BDNF levels has been varying, both serum and plasma samples were analyzed in purpose of examining the consistency between serum and plasma findings.

The serum BNDF levels in the present patients remained quite constant but were remarkably higher than plasma BDNF. Five earlier studies have likewise reported no significant effect of ECT on serum BDNF levels (Fernandes et al., 2009; Gedge et al., 2012; Grønli et al., 2009; Kleimann et al., 2014; Rapinesi et al., 2015), but several others have reported an increase (Bilgen et al., 2014; Bocchio‐Chiavetto et al., 2006; Haghighi, Salehi, et al., 2013; Hu et al., 2010; Okamoto et al., 2008; Piccinni et al., 2009; Salehi et al., 2016). A recent review and meta‐analysis found an overall increase in blood BDNF levels after ECT series (Brunoni, Baeken, Machado‐Vieira, Gattaz, & Vanderhasselt, 2014). A major difference between these studies and the present study is the difference in the timing of blood sampling. Increase in serum or plasma BDNF levels was measured during the treatment period (Bilgen et al., 2014; Marano et al., 2007); in other studies, timing of the sampling varied from 1 day after the ECT series (Bilgen et al., 2014; Hu et al., 2010) to 1 month after the last ECT session (Bilgen et al., 2014; Bocchio‐Chiavetto et al., 2006; Bumb et al., 2014; Haghighi, Salehi, et al., 2013; Hu et al., 2010; Marano et al., 2007; Okamoto et al., 2008; Piccinni et al., 2009; Salehi et al., 2016).

Decreased serum BDNF levels have also been reported after ECT session (Stelzhammer et al., 2013). In the present study, the blood samples were taken during the whole ECT series; at baseline, 2, and 4 hr after ECT at the first, fifth, and last ECT session. Thus, the different timing of these studies makes them difficult to compare with the present one. The study by Grønli et al. (2007) resembles the present one most closely. In that study, serum BDNF levels were measured during the ECT series at baseline and five, 15, 30, and 60 min after the first, fourth, and eighth ECT session. No change was found in serum BDNF levels (Grønli et al., 2009). In the present study, the association between remission after ECT and both serum and plasma BDNF levels was analyzed. No associations were found between any plasma BDNF levels and remission.

Reports of associations between the changes in serum BDNF and outcome of ECT have on the whole been contradictory (Pinna et al., 2016). Hu et al. (2010) reported significant association between the elevation of serum BDNF level and a decreasing rate of MDD symptoms (Hu et al., 2010). Okamoto et al. (2008) reported a rise in serum BDNF level in responders, whereas, in nonresponders’ serum, BDNF levels remained unchanged (Okamoto et al., 2008). On the contrary, many other researchers found no correlation between serum BDNF levels and alleviation of depressive symptoms (Bilgen et al., 2014; Bocchio‐Chiavetto et al., 2006; Bumb et al., 2014; Salehi et al., 2016). Accordingly, in one study, ECT and aerobic exercise training (BDNF also existing in the muscle tissue) were more effective in reducing depressive symptoms than either ECT or aerobic exercise training alone. Also, this study found no association between the plasma BDNF levels and depression (Archer, Josefsson, & Lindwall, 2014; Salehi et al., 2016). These negative findings are consistent with the outcome of review and meta‐analysis by Brunoni et al. (2014) (Brunoni et al., 2014). In the present study, neither serum nor plasma BDNF levels predicted the later remission rate after the ECT, whereas, in some previous studies, a higher baseline level of serum BDNF was associated with treatment response of antidepressant medication (Mikoteit et al., 2014, 2016). Different mechanisms of the therapeutic action between ECT and antidepressant medications may explain the differences in the changes in BDNF levels between these treatment modalities. The effect of ECT mainly concerns other signaling systems than serotonin thus exerting less influence on BDNF levels (Huuhka et al., 2007, 2008).

A fall in the plasma BDNF levels of the present patients was observed during the fifth ECT session between the baseline and the 2‐hr samples. Otherwise, no influence of ECT on plasma BDNF levels was found. Earlier studies have reported no such decrease in plasma BDNF during or after ECT (Haghighi, Salehi, et al., 2013; Lin et al., 2013; Marano et al., 2007; Piccinni et al., 2009). Instead, some of these studies (except (Lin et al., 2013) reported increase in plasma BDNF in some point of treatment.

Several studies have reported the effects of various external factors on serum and plasma BDNF levels suggesting that BDNF is sensitive to external factors causing high inter‐ and intraindividual variations (Bus et al.., 2011; Chan, Tong, & Yip, 2008; Cubeddu et al., 2011).

Several studies have been published concerning BDNF levels and ECT. Four studies measured plasma BDNF levels (Haghighi, Salehi, et al., 2013; Lin et al., 2013; Marano et al., 2007; Piccinni et al., 2009), and twelve measured serum (Bilgen et al., 2014; Bocchio‐Chiavetto et al., 2006; Bumb et al., 2014; Fernandes et al., 2009; Gedge et al., 2012; Grønli et al., 2009; Hu et al., 2010; Kleimann et al., 2014; Okamoto et al., 2008; Rapinesi et al., 2015; Salehi et al., 2016; Stelzhammer et al., 2013). Both significant change and no change in BDNF levels after ECT have been reported with either method. The results appeared not to be related to whether plasma or serum was used. Furthermore, the findings from serum and plasma studies have often been discussed together despite the different methods. When comparing the serum and plasma BDNF levels of the present study, the correlations were random (0.187–0.636, Pearson correlation coefficient). Thus, serum and plasma levels of BDNF might not be comparable. When assessing the findings of the clinical studies regarding MDD and ECT in a meta‐analysis, Polyakova et al. (2015) reported an increase in plasma BDNF levels but not in serum. Neither plasma nor serum BDNF levels did associate with the response of ECT (Polyakova et al., 2015).

Elfving, Plougmann, and Wegener (2010) recommended that BDNF should be measured in serum since plasma may be influenced by the preanalytical processing of the blood sample. A meta‐analysis of BDNF in MDD reported greater standard deviations in plasma BDNF levels compared to serum BDNF which was also the case in the present study. Nevertheless, the number of studies evaluating plasma BDNF and MDD was small; thus, no recommendations could be made regarding the optimal method for measuring BDNF in blood (Brunoni, Lopes, & Fregni, 2008).

Brain‐derived neurotrophic factor is mainly stored in human platelets, from which it is released through platelet activation and degranulation of platelets during the clotting process. Therefore, serum BDNF levels have been reported to be remarkably higher (approximately 10–27 ng/ml) than those in plasma (Fujimura et al., 2002; Karege et al., 2005; Yamamoto & Gurney, 1990). The plasma levels in the present study were likewise considerably lower than the serum levels.

The strengths of the study include standardized timing of blood sampling during the individual ECT session and throughout the ECT series. One prior study has also measured the BDNF levels with the resembling methodology (Grønli et al., 2009). The present study is a preliminary research since as far as we know no other study has compared serum and plasma BDNF levels during ECT series.

A limitation of the study is the relatively small number of patients. Post hoc power analysis showed that a sample of altogether 128 patients would have been required to detect a difference of 0.5 or greater (≥4 ng/ml) in effect size between remitters and nonremitters with a power of 0.8. Therefore, the small sample size may explain the mainly negative findings. Another limitation is the assumption that serum or plasma BDNF levels would directly reflect BDNF levels in the brain. Kyeremanteng, James, Mackay, and Merali (2012) reported no clear correlation between brain and serum BDNF after ECS (Kyeremanteng et al., 2012). BDNF expression in different brain areas as well as between CSF and serum has been reported to have temporal variation following ECS (Angelucci et al., 2002; Kyeremanteng et al., 2012, 2014). Peripheral BDNF appears to go through specific differential regulation after ECT (Bilgen et al., 2014; Bocchio‐Chiavetto et al., 2006; Bumb et al., 2014; Haghighi, Salehi, et al., 2013; Hu et al., 2010; Okamoto et al., 2008; Piccinni et al., 2009; Salehi et al., 2016; Stelzhammer et al., 2013). A time delay between brain tissue and serum BDNF levels has been reported in both human and rodent studies (Bumb et al., 2014; Sartorius et al., 2009). Also, since the patients were given psychotropic medication during the study and lacking a control group, excluding the plausible effect of medication on serum and plasma BDNF levels is unfeasible. However, the possible effect of psychotropic medications assumedly remained stable since they were kept unchanged throughout the study.

In conclusion, the main finding of this study was that either serum or plasma BDNF levels were not associated with remission after ECT series in MDD. However, the plasma BDNF levels decreased after the fifth ECT session. Thus, predictive value of BDNF for effects of ECT remains uncertain. Also, the serum and plasma BDNF levels did not appear to be consistent with each other suggesting thus the separate methods are not comparable.

## CONFLICT OF INTERESTS

None declared.
